# Supplementary material for: Knowledge, attitude and practices of community health workers on managing and preventing childhood malaria and diarrhea in Fako Division, South West Region, Cameroon; A mixed method study
Source: PLOS Glob Public Health. 2023 Feb 21;3(2):e0001093. doi: 10.1371/journal.pgph.0001093 (PMC10021294; doi:10.1371/journal.pgph.0001093)
Supplement: S1 Table — (DOCX) [file pgph.0001093.s001.docx]

## Transcription templates for Focus Groups Discussions

## (CHWs of Tiko Health District)

## UNIVERSITY OF BUEA

**The Knowledge, Attitudes and Practices (KAP) of community health workers regarding management of malaria and childhood diarrhea in Fako Division South-West Region, Cameroon**

**Participant ID:** KAP_MCD_FGD_CHW_001_CNN

**Key**: KAPMCD

**FDG**= DATA COLLECTION METHOD

**CHW=**WITH COMMUNITY HEALTH WORKERS

**001**-SERIAL

**CNN**= CHI NDUM NAOMI (TRANSCRIBER)

**Date of Interview:** 6/07/2020/FGD

**Duration of each Interview**: 45_mins

**Data Collection Method**: FOCUS GROUP DISCUSSION

**Start:** 10:15AM **End:** 11:00AM

**Name of Interviewer/ Facilitator/Data Collector:** Chi Ndum Naomi

**Translator (If appropriate):**

**Location of the interview/data collection method:** TDHS/ FGD

**Observations from the interview/FGD:**

The participants were very interactive, willing to participate and to share information. During the discussion some participants where actively participating at all time while others were not very active. The discussion took place TDHS. Participants were arranged to sit on benches. The environment was relatively calm with very little or no distractions.

**Main body of transcript:**

F: **Have you ever received training on malaria or childhood diarrhea management?**

**P 1**: “Some (a few) of us were selected and given training on how to manage malaria. We were taught how to do the RDT and were told that we will be given test strips drugs and other items which we could use in the community to manage malaria at the level of the community. Till today nothing has been given to us. It’s been more than five years now; we are still waiting. Yes, we have attended seminars about diarrhea”

P 4: “A few CHWs were, but I wasn’t one of them.

P 9: “No, I have not been trained.”

P 2: “No I have not been trained to manage neither malaria nor diarrhea.

P 7: “Management of malaria and childhood diarrhea is not in our package of activities as CHWs.”

-Yes some of us were trained 4-5years ago.

-No, I have not.

-It’s not part of or minimum package of activities

**What is diarrhea?**

**P** 1: “dehydration”

P 3: “vomiting”

P 4: “Watery stool”

**P 6: “frequent stools of 3 or more than times a day and the stool is usually slimy in nature”**

**Definition of diarrhea**

**-**Frequent stools of greater than three times a day

-Dehydration

-Vomiting

Researcher: ‘” diarrhea is actually the frequent passage of watery stool of three or more than three times a day. There are three types of diarrhea. The most different is the diarrhea mixed with blood which is called dysentery.”

**F: What do we think causes childhood diarrhea?**

**Cause of diarrhea**

P 1: “Teething when the child’s teeth starts coming out.”

P 2: “Poor nutrition”

P 3: “when the child starts eating solid food.”

P 4: “Poor hygiene condition”

Researcher: Poor hygiene and sanitation is the main cause of childhood diarrhea because it starts at the center of the activities of a breastfeeding mother. Right from the way she cares for her baby to the way she prepares her food, to the precautions she takes before and while feeding the child. This is the health education CHWs are expected to give breasting feeding mothers and community members.

**F: How will you recognize a child suffering from diarrhea what are the signs and symptoms?**

P 1: “dehydration”

P 2: “number of stools equal to or more than 3 times in a day, loss of petite”

P 4: “Weight loss”

P 6: “Body weakness”

Researcher: “In children we will commonly have dehydration, body weakness, weight loss, dry skin, cracked lips, all coupled with stools greater than or equal to three times a day.

**Sign and Symptoms of childhood diarrhea**

-Dehydration

-Body weakness

-Weightless

-Greater than 3 stools a day

**F: Do you think diarrhea can be treated at home without going to the hospital?**

P 1: “Yes, childhood diarrhea can be treated at home without going to the hospital”

P 2: “Yes, childhood diarrhea can be treated at home without going to the hospital”

P 3: “Yes, childhood diarrhea can be treated at home without going to the hospital”

P 5: “Yes, childhood diarrhea can be treated at home without going to the hospital”

P 7: “Yes, childhood diarrhea can be treated at home without going to the hospital”

P 8: “Yes, childhood diarrhea can be treated at home without going to the hospital”

**Childhood diarrhea can be successfully treated at home**

-Yes, diarrhea can be treated at home.

**F: What are the things you will do to manage a child suffering from diarrhea?**

P 1: “I will prepare and give the child oral rehydration salt solution and then observe the child.”

P 3: “Sometimes I give fresh (young) guava leaves and I advise children to chew.”

P 5: “I usually prepare and give ORS to the child and I also give zinc supplements.”

P 6: “Sometimes I mix charcoal with salt and red oil and give to the child.”

**Treatment for childhood diarrhea**

**-**Prepare and administer ORS

-Give guava leaves

- Give a mixture of ground charcoal with red oil and salt

**F: Will you advise that traditional herbs be used to manage childhood diarrhea?**

P 1: “Yes, I will recommend that traditional herbs be used because of the unavailability of both ORS and zinc supplements in most health areas and even in health facilities. Another reason why I will recommend the use of traditional herbs because it is cheap and affordable. No cost of transportation we do not need to buy the leaves we use and we do not need to pay transport to go and buy them. And also because they are accessible”

P 2: “Yes of course I will advise people to use traditional helps in treating childhood diarrhea. Because I have used it and it is really effective. Sometimes, the health area is far from the town, in such cases, I will recommend people in that community to use the traditional herbs that works for them”

P 4: “Yes, I will recommend the use of traditional herbs in managing diarrhea in children because it is less expensive.”

Recommend traditional herbs as treatment of childhood diarrhea

**-**I will recommend the use of traditional herbs in the treatment of childhood diarrhea because it is cheap, easily accessible and effective.

**F: How do you prepare ORS?**

P 1: “you add 1 sachet of ORS into one litter of clean water.”

P 4: “By adding 1 sachet of ORS into 1.5L of clean water.”

P 6: “By adding 1 sachet of ORS into 1.5L of clean water.”

P 9: “By adding 1 sachet of ORS into 1.5L of clean water.”

**Preparation of ORS**

-Add 1 sachet of ORS in 1 litter of water

- Add 1 sachet of ORS in 1.5 litter of water

**F: How do you prepare homemade salt sugar solution?**

P 1: “We add 8-10 cubes of sugar into 1L of clean water with half teaspoon of salt.”

P 4: “We add 5 cubes of sugar into 1L of clean water with one teaspoon of salt.”

P 5: “We add 10 cubes of sugar into 1L of clean water with one teaspoon of salt.”

P 8: “We add 5 cubes of sugar into 1.5 L of clean water with one teaspoon of salt.”

**Preparation of homemade salt sugar solution**

Add 8-10 cubes of sugar, half teaspoon of salt into 1l of water.

Adding 5 cubes of sugar, one teaspoon of salt into 1 litter of water.

Adding 10 cubes of sugar, one teaspoon of salt in 1L of water.

Add 5 cubes of sugar, one teaspoon of salt into 1.5L of clean water

**F: How often do you administer ORS/SSS to a child suffering from diarrhea and in what quantity?**

P 1: “A child should be given fluids more often that his usual intake. The quantity of fluid or ORS will depend on the severity of the diarrhea.”

P3: “More often than usual.”

P 7: “a child should be given fluids more often that his usual intake.”

**Frequency of administration of ORS**

-More than usual depending on the severity of the diarrhea.

-More than usual

**F: What advise will you give a breastfeeding mother about preventive measures for diarrhea?**

P 1: “Exclusive breastfeeding. Where she feeds the child only with breast milk for the first 6months.”

P 2:” Good hygiene and sanitation. She should always wash her hands and clean her breast/nipple before breastfeeding the child. For older babies the mother should give them clean or mineral water for drinking.”

P 3: “Exclusive breast feeding meaning only breastmilk everyday”.

P 4:” To always press and throw away the first breastmilk before breastfeeding the child.”

P 5: “Exclusive breastfeeding. Where she feeds the child only with breast milk for the first 6months. Without giving any water”

**Preventive measures against childhood diarrhea**

**-**Exclusive breastfeeding

-Good hygiene sanitation

-Personal hygienic practices

**F: What causes malaria?**

P 1: “The female *Anopheles* mosquito”

P 3: “Mosquito”

**Causes of malaria**

-Female Anopheles mosquito

-Mosquito

**F: How is malaria transmitted**

P 1: “When an infected mosquito bites another person”

P 4: “When a mosquito bites and infected person, it becomes infected then it bites a healthy person”

P 5: “Through the bite of a female *Anopheles* mosquito.”

**Transmission of malaria**

-Bite from an infected mosquito

-Bite of an infected female *Anopheles* mosquito

**F: What are the symptoms of simple malaria?**

P 1: “fever, body weakness, and broken joints paints”

P 7: “fever, body weakness, and broken joints paints”

P 9: “loss appetite”

**Signs and symptoms of malaria**

-Fever, body weakness and joint pains

-loss of appetite

**F: As a CHW how do you manage a patient with simple malaria?**

P 1: “I do the RDT on a suspected malaria patient and if he/she tests positive I then give an antimalarial medication.”

P 7: “I refer the patient to the hospital”

P 8: “Sometimes I refer the patient, sometimes I use traditional herbs. It depends on the patient’s state.”

**Manage of simple malaria**

**-**Do RDT, then administer antimalarial drugs to any positive case.

-Refer the patient to the hospital.

-Treat with traditional herbs

**F: How long do we treat a patient for simple malaria?**

P 1: “three days”

P 2: “three days”

P 3: “three days”

P 4: “three days”

P 6: “three days”

P 7: “three days”

**Length of treatment for simple malaria**

**-**Three days

**F: What are the common malaria treatment we prescribe to patients?**

P 1: “Artemether”

P 2: “Artesunate”

P 6: “Paracetamol”

P 8: “I use traditional herbs. Where I mix guava leaves, pawpaw leaves fever grass, black jar, moringa. Which I boil and drink.

P 9: “use back of trees which I boil and drink for 3-5 days.”

**Common malaria treatments**

-Artemether

- Artesunate

-Paracetamol

-Traditional herbs

**F: As a CHW how do you manage a case of severe malaria?**

P 1: “I immediately refer the patient to the hospital.”

**Management of severe malaria**

**-**immediately refer the patient to the hospital.

**F: What preventive measures will advise someone to consider for malaria?**

P 1: “Sleep under treated mosquito nets, empty potholes with standing water, and use mosquito repellants.”

P 3: “Sleep under mosquito nets’

P 5: “clear bushes around the house”

P 7: “No standing water around the house.”

**Preventive measures for malaria**

**-**Sleep under treated mosquito nets

-Clear bushes around the house

-Empty potholes

-Use mosquito repellants

**F: What is the first line treatment for malaria in Cameroon?**

No answer

**First line treatment for malaria in Cameroon**

-No answer

**(CHWs of Limbe Health District)**

**UNIVERSITY OF BUEA**

**The Knowledge, Attitudes and Practices (KAP) of community health workers regarding management of malaria and childhood diarrhea in Fako Division South-West Region, Cameroon**

**Participant ID:** KAP_MCD_FGD_CHW_002_CNN

**Key**: KAPMCD

**FDG**= DATA COLLECTION METHOD

**CHW=**WITH COMMUNITY HEALTH WORKERS

**002**-SERIAL

**CNN**= CHI NDUM NAOMI (TRANSCRIBER)

**Date of Interview:** 9/07/2020/FGD

**Duration of each Interview**: 40_mins

**Data Collection Method**: FOCUS GROUP DISCUSSION

**Start:** 9:00AM **End:** 9:40AM

**Name of Interviewer/ Facilitator/Data Collector:** Chi Ndum Naomi

**Translator (If appropriate):**

**Location of the interview/data collection method:** CMALIMBE/ FGD

**Observations from the interview/FGD:**

The participants were very interactive, willing to participate and to share information. During the discussion some participants where actively participating at all time while others were not very active. The discussion took place BCTC. Participants were arranged to sit on a round table. The environment was relatively calm with very little or no distractions.

**Main body of transcript:**

**F: Have we been trained to manage malaria and childhood diarrhea?**

P 1: “Yes, about 4-5 years ago some of us were selected and trained on how to do RDT and manage malaria at the level of our communities and we were given some antimalarial drugs which have finished. We no longer have malaria drugs.”

P 4: “We have been trained only on malaria alone, we’ve not received any formal training on diarrhea.”

P 5: “Some of us were trained on how to do RDT but that was about 5 years ago. But we were not given materials to work with, and since then nothing has been done.”

P 6: “We have been thought about malaria but we’ve not been trained on how to manage it. For diarrhea we just learn about it in the community.”

P 9: “we have been trained to manage malaria but we have not been given the materials to use.”

**Trained for management of malaria and childhood diarrhea**

**-** Yes some of us were trained to manage malaria 4-5years ago.

-We have been thought about malaria but not on how to manage it.

**F: What is diarrhea?**

P 1: “Watery stool”

P 3: “weight loss”

P 4: “Sunken eyes”

P 7: “diarrhea”

P 5: “Stomach ache”

P 11: “watery stool”

P 8: “Diarrhea is stool mixed with blood”

**Definition of Diarrhea**

-Watery stool

-Weight loss

-Sunken eyes

-Stomach ache

-Stool mixed with blood

**F: What are the signs and symptoms of childhood diarrhea?**

P 1: “Sunken eyes”

P 3: “By measuring the mid upper arm circumference.”

P 5: “The child’s eyes will be white and his body will feel weak and he will have fever.”

P 6 “We can measure the mid upper arm of the child”

P 8: “The child will look pale”

**Signs and Symptoms**

-Sunken eyes

-Measuring the mid upper arm circumference

-White eyes

-Body weakness

-Fever

**F: What do you do to a child suffering from diarrhea in your community?**

P 2: “We give ORS”

P 5: “in my case, I boil water, add a little bit of salt and a little bit off sugar. For example, for one litter of water, I add ½ teaspoon of salt with 6 cubes of sugar inside, I shake it then give it to the child. After giving this to the child, I look for herbs like guava leaves squeeze it to extract the juice then give a teaspoon of the juice to the child. Sometimes, I use traditional herbs like macepo leaves which I squeeze out the juice add a little bit of salt, stir and give ¼ tea cup to the child. I do this to children >5 years. I can then take the child to the hospital if the diarrhea persists”

P 6: “By giving the ORS solution to the child.”

P 9: “I give the child homemade salt sugar solution.”

**Management of childhood diarrhea**

-Administer ORS

-Give homemade salt sugar solution

-Give traditional herbs

**F: How do you prepare ORS?**

P 2: “by mixing one sachet of ORS in one liter of water.”

P 4: “By adding it into water.”

P11: “By mixing one sachet of ORS in 1liter of water.”

**Preparation of ORS solution**

-Adding 1 sachet of ORS into 1 liter on water

-Adding ORS in water

**F: How do you prepare the homemade salt sugar solution?**

P 1: “Mixing I teaspoon of salt 5 cubes of sugar in 1 liter of water”

P 2: “I teaspoon of salt 5 cubes of sugar in 1 liter of water”

P 4: “My mixing by mixing salt and sugar in water”

P 5: “For one litter of water, I add ½ teaspoon of salt with 6 cubes of sugar inside”

P 7: “By mixing 1 teaspoon of salt in 1.5 liters of water plus 8 cubes of sugar.”

P11 “By mixing 1 teaspoon of salt in 1.5 liters of water plus 8 cubes of sugar.”

**Preparation of salt sugar solution**

-Mixing 1 teaspoon of salt, 5 cubes of sugar in 1L of water.

-Mixing salt, sugar and water

-Mixing 1 teaspoon of salt, 8 cubes of sugar into 1.5L of water

-Mixing ½ teaspoon of salt, 6 cubes of sugar into 1L of water

-Mixing 1 teaspoon of salt, 8 cubes of sugar inti 1.5L of water.

**F: what are the preventive measures for diarrhea that you will advise a breastfeeding mother?**

P 11: “Advise the mother to carryout exclusive breastfeeding for a period of six months.”

P 5: “By practicing good hygiene and sanitation”

**Preventive measures for diarrhea**

-Good hygiene and sanitation

-Exclusive breastfeeding

**F: What causes malaria**

P 2: “Infected female Anopheles mosquito”

P 5: ‘Mosquito bite”

P 7: “Female *Anopheles* mosquito”

P 8: Female *Anopheles* mosquito”

P 10: “Anopheles mosquito”

**Cause of malaria**

-Infected female *Anopheles* mosquito

-Female Anopheles mosquito

- Anopheles mosquito

**F: How do mosquitoes get infected?**

P 2: “By biting individuals who are already infected.”

P 5: “From contaminated water.”

P 8: “Mosquitoes are born already infected.”

P 11: “Mosquitoes get the infection after biting an infected person.”

**How mosquitoes get infected**

-By biting individuals already infected

-From contaminated water

-They are born already infected

**F: What is the signs and symptoms of malaria?**

P 1: “high temperature”

P 2: “Vomiting”

P 3: “Headache”

P 7: “chills”

P 9: “vomiting and very high temperature.”

**Signs and Symptoms of simple malaria**

-high temperature

-Vomiting

-Headache

-Chills

**F: What do you do when you identify a suspected case of malaria?**

P 1: “I refer the patient to the health center”

P 4: “Sometimes I refer the patient to the hospital, but sometimes I tell them to buy antimalarial drugs like Arthemeter”

P 5 “In my own case, I do not refer the patient immediately. Usually, what I do is I encourage the use of traditional herbs. A mixture of paw-paw leaves, guava leaves mango leaves, ‘masepo’, fever grass and other which I boiled very well then I drink in a tea cup, I also and cover myself with it very morning and evening. I’ll do this for three days. On the third day of the treatment, I heat the remaining treatment then use it to “pump” myself with. I do this for both myself and my kids. If the malaria persists, I then go to the hospital.

**Management of simple malaria**

-Refer to the hospital

-Prescribe antimalarial medications

-Use traditional herbs

**F: What is the first line treatment for malaria in Cameroon?**

P 1:” Artemether”

P 2: “Artemether”

P 3: “Paracetamol”

P 6: “Bimalaria”

P 8: “Arthemeter”

P 9: “Quinine Sulphate”

P10: “Arthemeter”

**First-line treatment for malaria in Cameroon**

-Artemether

-Paracetamol

-Bimalaria

-Quinine Sulphate

**F: What are the preventive measures against malaria?**

P 2: “Good hygienic conditions”

P 5: “emptying potholes around the house”

P 6: “Spray the house with insecticides”

P 7: “Clearing of bushes around the house.”

P 8: “sleeping under insecticide treated mosquito nets.”

**Preventive measures against malaria**

-Good hygiene conditions

-Empty potholes

-Use insecticide sprays

-Clear bushes around the house

-Sleep under treated mosquito nets

# (CHWs of Buea Health District)

**UNIVERSITY OF BUEA**

**The Knowledge, Attitudes and Practices (KAP) of community health workers regarding management of malaria and childhood diarrhea in Fako Division South-West Region, Cameroon**

**Participant ID:** KAP_MCD_FGD_CHW_003_CNN

**Key**: KAPMCD

**FDG**= DATA COLLECTION METHOD

**CHW=**WITH COMMUNITY HEALTH WORKERS

**003**-SERIAL

**CNN**= CHI NDUM NAOMI (TRANSCRIBER)

**Date of Interview:** 9/07/2020/FGD

**Duration of each Interview**: 47_mins

**Data Collection Method**: FOCUS GROUP DISCUSSION

**Start:** 10:10AM **End:** 11:57AM

**Name of Interviewer/ Facilitator/Data Collector:** Chi Ndum Naomi

**Translator (If appropriate):**

**Location of the interview/data collection method:** BDHS/ FGD

**Observations from the interview/FGD:**

The participants were very interactive, willing to participate and to share information. During the discussion some participants where actively participating at all time while others were not very active. The discussion took place BCTC. Participants were arranged to sit on a round table. The environment was relatively calm with very little or no distractions.

**Main body of transcript:**

**F: Have you ever received training on the management of malaria and childhood diarrhea?**

P 2: “Even though some of us were selected and trained to manage malaria, we were not given materials to work with. But I have also received training from an NGO called Relief International. There, I was trained to manage malaria, diarrhea and pneumonia, and I was given the kits to manage these ailments.”

P 4: “some of us have been trained on malaria but that was about five years ago, since then we have not had any trainings. CHWs who were recruited since this time have not had training.

P 7: “I and a few CHWs were selected to be and trained to manage malaria. We were thought on how to do RDT but we were not given kits to manage cases in the community.”

P 6: “I have not received training on malaria nor diarrhea.”

P 8: “We have attended seminars for both malaria and diarrhea, but were not thought how to manage any of the diseases as CHWs.”

**Trained to manage malaria and childhood diarrhea**

-some of us were selected and trained to manage malaria but that was about 5 years ago

-Recently recruited CHWs have not been trained

-Attended seminars on the diseases but we have not received any formal training

**F: What is diarrhea?**

P 1: “Diarrhea is purging.”

P 2: “Diarrhea is purging which occurs more than 3 times in a day.”

**Definition of diarrhea**

-Diarrhea is purging

-Purging which occurs more than 3 times a day

**F: What are the signs and symptoms of diarrhea in children?**

P 1: “watery stool”

P 2: “stool more than three times a day, stool mixed with blood.”

P 3: “weight loss”

P 6: “loss of appetite”

P 8: “body weakness”

P 9: “Fever”

**Signs and Symptoms**

-watery stool

-Stool greater than 3 times a day

-Weight loss

- Loss of appetite

- body weakness

- Fever

**F: How do you prepare homemade ORS?**

P 3: “The ORS comes in its packet with tablets. So we add one sachet of ORS into 1 liter of clean water.”

P 5: “By adding one sachet of ORS in one litter of water.”

P 9: “The ORS comes in its packet with tablets. So we add one sachet of ORS into 1 liter of clean water.”

**Preparation of ORS solution**

Adding one sachet of ORS into 1L of water

**F: How do you prepare homemade salt sugar solution?**

P 2: “half teaspoon of salt, five cubes of sugar in one litter of water.”

P 4: “I do not know.”

P 9: “one level teaspoon of salt, five cubes of sugar in one litter of water the you shake until the mixture is uniform”

**Preparation of homemade salt sugar solution**

-I do not know

-Mixing 1 level teaspoon of salt, 5 cubes of sugar in 1L of water.

-Mixing ½ teaspoon of salt, 5 cubes of salt in 1L

**F: Traditionally, what do you do in managing diarrhea?**

P 2: “I use fresh guava leaves and it works for them. I squeeze the leaves to extract the juice out of the leaves and they give it to the child.”

P 4: “I do use the fresh guava leaves on both children and adult. I can’t be precise about the dose, but for adults chew the fresh guava leaves while for children we boil the leaves and give the child one or two teaspoons three times a day to the child and before you know it the child is find again.”

P 6: “I use fresh guava leaves to manage diarrhea, but usually not in children.”

P 8: “I do use fresh guava leaves to manage diarrhea in my home both for myself and my kids.”

**Traditional management of diarrhea**

-Using extracted fresh guava leaves juice

**F: What causes malaria??**

P 1: “Female *Anopheles* mosquito”

P 2: “Malaria is caused by the female *Anopheles* mosquito.”

P 3: “the female *Anopheles* mosquito.”

P 6: “The female *Anopheles* mosquito causes malaria.”

**Causes of malaria**

-Female *Anopheles* mosquito

**F: How do mosquitoes get infected?**

P 1: “When they bite someone who is already infected.”

P 2:” They are born infected.”

P 4: “Mosquitoes are born infected.”

P 9: “Mosquitoes are born already infected.”

**How mosquitoes get infected**

-By biting someone already infected

-They are born infected

**F: What do you do when you identify a case of malaria in the community?**

P 2; “I worked with Relief international where they give me drugs an all materials to manage malaria. Which use in the community”

P 3: “I immediately refer him/her to the nearest hospital or health center.”

P 4: “After our training we used to be given Artesunate amodiaquine (AZAC) which we used to manage malaria in the community, but for about one year now we are no longer receiving these drugs from the Drug Funds.”

P 5: “I just refer the patient to the hospital.”

P 8: “I prescribe anti malaria drugs to some and I refer cases which I consider serious to the hospital.”

P 9: “I advise the patients to use traditional herbs”

**Management of Simple malaria**

-Do RDT and administer antimalarial medications

-Refer to the hospital/health center

-Prescribe antimalarial medications

- Advice the patient to use traditional herbs

**F: Traditionally, what do you do to manage cases of malaria?**

P 4: “I have members in my community who use what we call “dogo yaro” leaves which they slice and put inside a litter of a strong alcoholic drink called “afofo” and they allow it to ferment for three days. They take one shot in the morning and one hot in the evening and it works for them.”

P 5: “I mix paw-paw leaves, lemon, black jack, back of mango tree, back of pear tree and boil. Allow the mixture to get cold then I sifter and put in in 1/.5 liter containers hen I give one glass of the mixture to the patient in the morning and another glass in the evening for five days. Using the heat from the pot of boiled mixture, sometimes I encourage the patient to cover himself with a thick cloth/blanket to hasting the healing process.

P 7: “I boil paw-paw leaves with moringa and give the mixture to the patient, one glass in the morning and another glass in the evening.”

P 9: “The thing is anything you believe works will work. With severe malaria, I use sand leaves, paw-paw leaves fever grass with lemon and “black jack” which I boil, sifter and put in 5 liter containers. I give the patient one glass of the mixture three times a day for seven days. Usually, I encourage the patient to use a blanket and cover himself inside the heat coming from the pot of the boiled mixture.”

**Traditional ways in managing of malaria**

-Boil a mixture of leaves and back of trees and administer of 3 days.

**F: What is the first line treatment for malaria in Cameroon?**

P 2: “Artemether”

P 4: “Artemether”

P 7: “Artesunate”

P 5; “Bimalaria”

**First line treatment of malaria in Cameroon**

-Artemether

-Artesunate

- Bimalaril

**F: What are some of the malaria preventive measures you will advise people to practice?**

P 1: “Clear bushes around the house, sleep under iTNs.”

P 2: “Empty potholes filled with water, use mosquito sprays.”

P 4: “Wear long sleeve clothes, clear bushes around the house.”

P 7: “Clean bushes around the house and under mosquito nets.”

P 9: “Wear long sleeve clothes”

**Preventive measures against malaria**

Clear bushes around the house

Wear long sleeve clothes

Empty potholes

Sleep under treated mosquito nets

# Table A: Thematic Analysis Focus Group Discussions

| **THEMES** | **TIKO** | **LIMBE** | **BUEA** | **PERTINENT CITIATIONS** |
| --- | --- | --- | --- | --- |
| TRAINED TO MANAGE MALARIA OR CHILDHOOD DIARRHEA | -Yes some of us were trained 4-5years ago.  -No, I have not.  -It’s not part of or minimum package of activities. | **-** Yes some of us were trained to manage malaria 4-5years ago.  -We have been thought about malaria but not on how to manage it. | -some of us were selected and trained to manage malaria but that was about 5 years ago  -Recently recruited CHWs have not been trained  -Attended seminars on the diseases but we have not received any formal training | Management of malaria and childhood diarrhea is not in our package of activities as CHWs**. (P 7, TIKO)**  We have been thought about malaria but we’ve not been trained on how to manage it. For diarrhea we just learn about it in the community**. (P 6, LIMBE)**  Even though some of us were selected and trained to manage malaria, we were not given materials to work with. But I have also received training from an NGO called Relief International. There, I was trained to manage malaria, diarrhea and pneumonia, and I was given the kits to manage these ailments. **(P2, BUEA)** |
| DEFINITION OF DIARRHEA | **-**Frequent stools of greater than three times a day  -Dehydration  -Vomiting | -Watery stool  -Weight loss  -Sunken eyes  -Stomach ache  -Stool mixed with blood | -Diarrhea is purging  -Purging which occurs more than 3 times a day | Frequent stools of 3 or more than times a day and the stool is usually slimy in nature. **(P 6, TIKO)**  Diarrhea is stool mixed with blood. **(P8, LIMBE)**  Diarrhea is purging which occurs more than 3 times in a day. **(P2, BUEA)** |
| SIGNS AND SYMPTOMS OF CHILDHOOD DIARRHEA | -Dehydration  -Body weakness  -Weightless  -Greater than 3 stools a day | -Sunken eyes  -Measuring the mid upper arm circumference  -White eyes  -Body weakness  -Fever | -watery stool  -Stool greater than 3 times a day  -Weight loss  - Loss of appetite  - body weakness  - Fever | The child’s eyes will be white and his body will feel weak and he will have fever. **(P 5, LIMBE)**  Number of stools equal to or more than 3 times in a day, loss of petite. **(P2, TIKO)**  The child will be dehydrated. **(P 1, TIKO)** |
| TREATMENT OF CHILDHOOD DIARRHEA | **-**Prepare and administer ORS  -Give guava leaves  - Give a mixture of ground charcoal with red oil and salt | -Administer ORS  -Give homemade salt sugar solution  -Give traditional herbs | Administer ORS  -Give homemade salt sugar solution | I will prepare and give the child oral rehydration salt solution and then observe the child. **(P 1, TIKO)**  Sometimes I give fresh (young) guava leaves and I advise children to chew**. (P 3 TIKO)**  Sometimes I mix charcoal with salt and red oil and give to the child. **(P 6, TIKO)**  In my case, I boil water, add a little bit of salt and a little bit off sugar. For example, for one litter of water, I add ½ teaspoon of salt with6 cubes of sugar inside, I shake it then give it to the child. After giving this to the child, I look for herbs like guava leaves squeeze it to extract the juice then give a teaspoon of the juice to the child. Sometimes, I use traditional herbs like macepo leaves which I squeeze out the juice add a little bit of salt, stir and give ¼ tea cup to the child. I do this to children >5years. I can then take the child to the hospital if the diarrhea persists. **(P 5, LIMBE)** |
| PREPARATION OF ORS |  | -Adding 1 sachet of ORS into 1 litter on water  -Adding ORS in water | -Adding one sachet of ORS into 1L of water | The ORS comes in its packet with tablets. So we add one sachet of ORS into 1 litter of clean water. **(P 9, BUEA)**  By mixing one sachet of ORS in 1litter of water. **(P 11, LIMBE)**  By adding 1 sachet of ORS into 1.5L of clean water. **(P 4, TIKO)** |
| PREPARATION OF HOMEMADE SALT SUGAR SOLUTION | -Add 8-10 cubes of sugar, half teaspoon of salt into 1l of water.  -Adding 5 cubes of sugar, one teaspoon of salt into 1 liter of water.  -Adding 10 cubes of sugar, one teaspoon of salt in 1L of water.  -Add 5 cubes of sugar, one teaspoon of salt into 1.5L of clean water. | -Mixing 1 teaspoon of salt, 5 cubes of sugar in 1L of water.  -Mixing salt, sugar and water  -Mixing 1 teaspoon of salt, 8 cubes of sugar into 1.5L of water  -Mixing ½ teaspoon of salt, 6 cubes of sugar into 1L of water  -Mixing 1 teaspoon of salt, 8 cubes of sugar inti 1.5L of water. | -I do not know  -Mixing 1 level teaspoon of salt, 5 cubes of sugar in 1L of water.  -Mixing ½ teaspoon of salt, 5 cubes of salt in 1L  -Using extracted fresh guava leaves juice | We add 8-10 cubes of sugar into 1L of clean water with half teaspoon of salt. **(P1, TIKO)**  For one litter of water, I add ½ teaspoon of salt with6 cubes of sugar inside. **(P 5, LIMBE)**  By mixing 1 teaspoon of salt in 1.5 liters of water plus 8 cubes of sugar**. (P 8, LIMBE)**  One level teaspoon of salt, five cubes of sugar in one litter of water the you shake until the mixture is uniform. **(P 9, BUEA)** |
| TRADITIONAL HERBS USED IN MANAGING CHILDHOOD DIARRHEA | **-**I will recommend the use of traditional herbs in the treatment of childhood diarrhea because it is cheap, easily accessible and effective. |  | -Using extracted fresh guava leaves juice | Yes, I will recommend that traditional herbs be used because of the unavailability of both ORS and zinc supplements in most health areas and even in health facilities. Another reason why I will recommend the use of traditional herbs because it is cheap and affordable. No cost of transportation we do not need to buy the leaves we use and we do not need to pay transport to go and buy them. And also because they are accessible**. (P1, TIKO).**  I do use the fresh guava leaves on both children and adult. I can’t be precise about the dose, but for adults chew the fresh guava leaves while for children we boil the leaves and give the child one or two teaspoons three times a day to the child and before you know it the child is find again. **(P 4, BUEA)** |
| PREVENTIVE MEASURES AGAINST CHILDHOOD DIARRHEA | **-**Exclusive breastfeeding  -Good hygiene sanitation  -Personal hygienic practices | -Good hygiene and sanitation  -Exclusive breastfeeding |  | Good hygiene and sanitation. She should always wash her hands and clean her breast/nipple before breastfeeding the child. For older babies the mother should give them clean or mineral water for drinking. **(P 2, TIKO)**  Exclusive breastfeeding. Where she feeds the child only with breast milk for the first 6 months. Without giving any water. **(P 5, TIKO)**  Advise the mother to carryout exclusive breastfeeding for a period of six months. **(P11, LIMBE)** |
| CAUSE OF MALARIA | -Female Anopheles mosquito  -Mosquito | -Infected female *Anopheles* mosquito  -Female Anopheles mosquito  - Anopheles mosquito | -Female *Anopheles* mosquito | Infected female Anopheles mosquito. **(P2, LIMBE)**  Malaria is caused by mosquitoes **(P 3, TIKO)**  Female *Anopheles* mosquito. **(P2, BUEA)** |
| TRANSMISSION OF MALARIA | -Bite from an infected mosquito  -Bite of an infected female *Anopheles* mosquito | Bite of an infected female *Anopheles* mosquito | Bite of a female *Anopheles* mosquito | When a mosquito bites and infected person, it becomes infected then it bites a healthy person. (P 4, TIKO) |
| SIGNS AND SYMPTOMS OF MALARIA | -Fever, body weakness and joint pains  -loss of appetite | -high temperature  -Vomiting  -Headache  -Chills |  | Fever, body weakness, and broken joints paints. (P 7, TIKO)  Headache is one of the main symptoms of malaria. **(P3, LIMBE)** |
| TREATMENT OF SIMPLE MALARIA | **-**Do RDT, then administer antimalarial drugs to any positive case.  -Refer the patient to the hospital.  -Treat with traditional herbs | -Refer to the hospital  -Prescribe antimalarial medications  -Use traditional herbs | -Do RDT and administer antimalarial medications  -Refer to the hospital/health center  -Prescribe antimalarial medications  - Advice the patient to use traditional herbs | I do the RDT on a suspected malaria patient and if he/she tests positive I then give an antimalarial medication. **(P1, TIKO)**  In my own case, I do not refer the patient immediately. Usually, what I do is I encourage the use of traditional herbs. A mixture of paw-paw leaves, guava leaves mango leaves, macepo, fever grass and other which I boiled very well then I drink in a tea cup, I also and cover myself with it very morning and evening. I’ll do this for three days. On the third day of the treatment, I heat the remaining treatment then use it to “pump” myself with. I do this for both myself and my kids. If the malaria persists, I then go to the hospital. **(P 5, LIMBE)**  After our training we used to be given Artesunate amodiaquine (AZAC) which we used to manage malaria in the community, but for about one year now we are no longer receiving these drugs from the Drug Funds. **(P4, BUEA)**  I worked with Relief international where they give me drugs an all materials to manage malaria. Which use in the community. (P2 BUEA)  I just refer the patient to the hospital. **(P11, LIMBE)**  I prescribe anti malaria drugs to some and I refer cases which I consider serious to the hospital. **(P8, LIMBE)**  Some (a few) of us were selected and given training on how to manage malaria. We were taught how to do the RDT and were told that we will be given test strips drugs and other items which we could use in the community to manage malaria at the level of the community. Till today nothing has been given to us. It’s been more than five years now; we are still waiting. Yes, we have attended seminars about diarrhea. **(P1, TIKO)**  Management of malaria and childhood diarrhea is not in our package of activities as CHWs. **(P7, TIKO)** |
| TRADITIONAL MANAGEMENT OF MALARIA | -Boil a mixture of leaves and back of trees and administer of 3 days. | -Boil a mixture of leaves and back of trees and administer of 3 days. | -Boil a mixture of leaves and back of trees and administer of 3 days. | The thing is anything you believe works will work. With severe malaria, I use sand leaves, paw-paw leaves fever grass with lemon and “black jack” which I boil, sifter and put in 5 litter containers. I give the patient one glass of the mixture three times a day for seven days. Usually, I encourage the patient to use a blanket and cover himself inside the heat coming from the pot of the boiled mixture. **(P 9, BUEA)**  I mix paw-paw leaves, lemon, black jack, back of mango tree, back of pear tree and boil. Allow the mixture to get cold then I sifter and put in in 1/.5 liter containers hen I give one glass of the mixture to the patient in the morning and another glass in the evening for five days. Using the heat from the pot of boiled mixture, sometimes I encourage the patient to cover himself with a thick cloth/blanket to hasting the healing process. **(P5, BUEA)**  In my own case, I do not refer the patient immediately. Usually, what I do is I encourage the use of traditional herbs. A mixture of paw-paw leaves, guava leaves mango leaves, macepo, fever grass and other which I boiled very well then I drink in a tea cup, I also and cover myself with it very morning and evening. I’ll do this for three days. On the third day of the treatment, I heat the remaining treatment then use it to “pump” myself with. I do this for both myself and my kids. If the malaria persists, I then go to the hospital. **(P5, LIMBE)**  I use traditional herbs. Where I mix guava leaves, pawpaw leaves fever grass, black jar, moringa. Which I boil and drink. **(P8, TIKO)** |
| FIRSTLINE TREATMENT FOR MALARIA IN CAMMEROON | Artemether  - Bimalaril | -Artemether  -Paracetamol  -Bimalaril  -Quinine Sulphate | -Artemether  -Artesunate  - Bimalaril | Artemether is the first line treatment for malaria. **(P2 BUEA)**  Quinine Sulphate is used in treating malaria **(P9, LIMBE)**  Bimalaril is one of the antimalaria drugs we use. **(P6, LIMBE)** |
| METHHODS OF MALARIA PREVENTION | **-**Sleep under treated mosquito nets  -Clear bushes around the house  -Empty potholes  -Use mosquito repellants | -Good hygiene conditions  -Empty potholes  -Use insecticide sprays  -Clear bushes around the house  -Sleep under treated mosquito nets | -Clear bushes around the house  -Wear long sleeve clothes  -Empty potholes  Sleep under treated mosquito nets | Sleeping under insecticide treated mosquito nets. **(P8, LIMBE)**  Wear long sleeve clothes, clear bushes around the house. **(P4, BUEA)**  Emptying potholes around the house. **(P5, LIMBE)** |
