# Supplementary material for: Knowledge, attitude and practices of community health workers on managing and preventing childhood malaria and diarrhea in Fako Division, South West Region, Cameroon; A mixed method study
Source: PLOS Glob Public Health. 2023 Feb 21;3(2):e0001093. doi: 10.1371/journal.pgph.0001093 (PMC10021294; doi:10.1371/journal.pgph.0001093)
Supplement: S1 Questionnaire — (DOCX) [file pgph.0001093.s003.docx]

## KAP Survey Questionnaire

**Knowledge, attitude and practices of community health workers on managing and preventing childhood malaria and diarrhea in Fako Division, South West Region, Cameroon; A mixed method study**

Thank you for accepting to be part of this study. Please give the best answer to help us carry out our research on the knowledge, attitude and practices regarding childhood malaria and diarrhea management and prevention by community health workers. Feel free to skip a question you do not feel comfortable answering.

Questionnaire Number………………….

Date of Interview……. /……. /……….

**Instruction:**

**MCQ;** Please Tick (√) the correct response

**Open ended questions**: write an answer on the space provided

**PART 1**

**SOCIO-DEMOGRAPHIC CHARACTERISTICS**

- 1. **How old are you (age in complete years)**

1**.**2 **SEX**: Male Female

1.3 Do you have any child age less than 5 years? A) Yes B) No

1.4 **How many children aged less than five (< 5 years) live in your household?**

1.5 **What is your marital status?**

Single Married Divorced Widow

1.6 **What is your highest level of education?**

No education Primary Secondary University

1.7 a) **Do you have any other occupation?**  A) YES B) NO

b) If **YES,** name it?

**1.8 Which health district do you work for?** Buea Tiko Limbe

**Part II**

**SECTION A: KNOWLEDGE OF CHILDHOOD MALARIA MANAGEMENT AND PREVENTION**

**1.1 What causes malaria?**

1. Not using a mosquito net
2. Housefly or mosquito
3. Poor hygiene and sanitation
4. Female Anopheles mosquito
5. Mosquito or a tsetse fly
6. Blood transfusion
   1. **How is malaria transmitted?**

A) Insect bite B) Wearing dirty clothes C) Mosquito bite D) Drinking dirty water

- 1. **Insects that transmit malaria bite most during? ………………………………….**

1. Day time B) Night Time C) Both during the day and at night D) I do not know
   1. **Which of these are common signs and symptoms for simple malaria?**
2. Loss of consciousness, headache, loss of appetite, joint pains
3. Fever, loss of consciousness, head ache, body pains
4. Fever, headache, loss of appetite, body pains.
5. Vomiting, fever and watery stool
   1. **What is a common symptom of a suspected case of** **severe malaria**? (**ONE ONLY)**
6. Vomiting within 24hours B) Loss of consciousness with temperature temperatures >37^o^c

C) Lack of appetite D) Difficulties in breathing with temperature temperatures >37^o^c

- 1. **a) Do you know about Rapid Diagnostic Test (RDT) for malaria)?** A) YES B) NO

1. **If** YES, **every suspected case of malaria should undergo a Rapid Diagnostic Test (RDT)**

A) I strongly agree B) I agree C) I do not agree D) I do not know

- 1. **All patients who test positive with RDT should receive the first dose of antimalarial treatment.**

1. I strongly agree B) I agree C) I do not agree D) I don’t know
   1. **How will you manage a patient who presents with symptoms of simple malaria?**
2. Refer to the patient immediately to the nearest Hospital.
3. Give paracetamol to treat fever.
4. Allow the patient to rest while I call for help.
5. Do rapid diagnostic test (RDT) and treat with anti-malaria drugs.
   1. **What is the first line treatment antimalaria drugs in Cameroon?**
6. Chloroquine
7. Artemisinin-combination therapy (ACT)
8. Amodiaquine
9. Quinine sulphate
10. I do not know
    1. **What are the two most common anti malaria drugs used in your community.**
11. Paracetamol B) Artesunate Amodiaquine C) Bactrim D) Eferalgan
12. Chloroquine F) Panadol G) Artemether Lumefantrine H) I don’t know

**1.11 A patient with positive rapid diagnostic test (RDT) for simple malaria receives treatment for number of days.**

A) 2days B) 1 days c) 3 days C) 1-week D) 2 weeks D) I don’t know

**1.12 What advice will you give a member of your community about mosquito nets**

1. Throw away after 6 months or when it gets dirty.
2. Mosquito nets are reusable; Wash and air-dry then reuse.
3. Unmount and never reuse an old mosquito net.
4. No Advice
5. I do not know

**SECTION B: ALTITUDE TOWARDS CHILDHOOD MALARIA MANAGEMENT AND PREVENTION**

**2.1 Insects that transmit malaria get the infection from?**

A) Biting individuals infected with malaria

B) Contaminated water

C) From the atmosphere

D) All mosquitoes are born already infected

E) I don’t know

**2.2 Sleeping under mosquito net prevent malaria.**

1. I strongly agree B) I agree C) I do not agree D) I don’t know

**2.3 Severe malaria can be caused by witchcraft.**

1. I strongly agree B) I agree C) I do not agree D) I don’t know
   1. **A mixture of boiled paw-paw, mango, fever grass leaves should be used as basic treatment for malaria at home.**
2. I strongly agree B) I agree C) I do not agree D) I don’t know
   1. **Pregnant women are more likely to be infected with malaria**
3. I strongly agree B) I agree C) I do not agree D) I don’t know
   1. **Every child <5 is supposed to do proactive screening for malaria.**
4. I strongly agree B) I agree C) I do not agree D) I don’t know

**2.7 Which group is mostly susceptible to malaria?**

1. Adults
2. Children < 5 years
3. Children < 5 years and pregnant women
4. Pregnant women
5. Everyone
6. I don’t know

**2.8 Malaria is a serious and life-threatening disease.**

A) YES B) NO C) I don’t know

**2.9 The poor are more likely to be infected with malaria than the rich.**

A) I strongly agree B) I agree C) I do not agree D) I don’t know

**2.10 From whom do you think people in your community prefer to get information and treatment for malaria?**

A) Traditional healers B) Community health workers C) Street drug vendors

D) Hospital E) Pharmacy I don’t know.

**2.11** **The use of mosquito nets is more important for pregnant women.**

A) I strongly agree B) I agree C) I do not agree D) I don’t know

2.12 **Mosquito nets make places to be hot that is why I do not use it at night.**

A) I strongly agree B) I agree C) I do not agree D) I don’t know

**2.13 RDT and treatment are recommended for every malaria case.**

A) I strongly agree B) I agree C) I do not agree D) I don’t know

**2.14 Malaria is a serious health problem in my community.**

A) I strongly agree B) I agree C) I do not agree D) I don’t know

**2.15 Side pain (Corner belle) is a consequence of malaria in children.**

A) I strongly agree B) I agree C) I do not agree D) I don’t know

**SECTION C: PRACTICES ON CHILDHOOD MALARIA MANAGEMENT AND PREVENTION**

3.**1 Have you ever managed a patient presenting with symptoms of malaria? A**) YES B) NO

3.**2 Have you ever performed a rapid diagnostic test (RDT) for malaria?** A) YES B) NO

**3.3 Do you administer treatment for simple malaria?** A) YES B) NO

**3.4 How often do you participate in the distribution of mosquito nets to in your community?**

A) Always B) Sometimes C) Never D) When I am the time E) I don’t know

**3.5 How often do you give health education on hygiene and sanitation to your community?**

1. Always B) Sometimes C) Never D) I don’t know

**3.6 How often do you prescribe malaria drugs for malaria cases in your community?**

1. Always B) Sometimes C) Never D) I don’t know

**b) If Yes, Name two antimalarial drugs you prescribe to your patients.**

**3.7 Do you have mosquito nets for distribution? A) Yes B) No**

3.8 **How often do you distribute them?** A) Always B) Sometimes C) Never D) I don’t know

**3.9 Which of these malaria preventive measures do you prefer?**

1. Clearing bushes around the farm B) Use insecticide spray

C) Use of mosquito net e) Both B and C

**3.10 In a day, how many injectable doses of first line malaria treatment is given to a child?**

A) Single dose B) Two doses C) Three doses D) Five doses E) I don’t know

**3.11 All Oral anti malaria drugs are administered alongside an antipyretic (against fever).**

A) I strongly agree B) I agree C) I do not agree D) I don’t know

**PART III**

**SECTION B: KNOWLEDGE OF CHILDHOOD DIARRHEA MANAGEMENT AND PREVENTION**

2.1 **What is diarrhea?**

A) Passage of loose or watery stool ones a day.

B) Passage of loose or watery stool more than 3 times a day.

C) Passage of hard stool more than three 3 times a day.

D) Passage of hard stool at least ones a day.

E) I do not know

F) “Purge belle”

**2.2 What causes diarrhea in children? (Choose all that apply)**

A) Poor nutrition B) Unsafe drinking water C) Unsafe fecal disposal

D) Witchcraft E) Intravenous drug use F) Teething G) I do not know

- 1. **Which conditions will prompt you to treat a child for diarrhea? ()**

1. Watery stool greater than 4 days B) Greater than four stools per day C) Weakness D) Sunken eyes E) Associated vomiting E) Weight loss F) Blood in watery stool G) Decrease in quantity of urine H) Fever cold hands and feet

I) I do not know………………………….

2.4 **Abstaining a baby from breast milk during diarrhea can prevent further episodes of diarrhea?** A) I strongly agree B) I agree C) I do not agree D) I do not know

2.**5 a) Do you know how to prepare Oral rehydration salt (ORS) solution?**

A) Yes B) No

**b)If Yes, how is it prepared? ………………………………………………………………..**

**c) If** Yes, **how often should Oral Rehydration Salt solution (ORS) be used as treatment for childhood diarrhea?**

A) Never B) Sometimes C) Always D) I do not know

**2.6 Zinc supplements are important in treating childhood diarrhea.**

A) I strongly agree B) I agree C) I do not agree D) I do not know

**2.7 Can be regarded as a consequence of childhood diarrhea.**

A) Loss of consciousness B) Weight loss C) Blood shortage D) Death E) I don’t know

2.8 **Where will you advice a mother to seek help if her child has diarrhea? (More than one answer is allowed)**

A) Pharmacy/drug seller B) Health Centre C) Hospital D) Traditional healer E) Community health worker F) Self-medication with herbs G) Self-medication with antibiotics H) Others ________________________________

**SECTION B: ATTITUDE TOWARDS CHILDHOOD DIARRHEA MANAGEMENT AND PREVENTION**

3.1 **Childhood diarrhea can be caused by witchcraft.**

A) I strongly agree B) I agree C) I do not agree

3.2 **Diarrhea is a disease of the poor.**

A) I strongly agree B) I agree C) I do not agree

3.3 **Traditional herbs are better for treating childhood diarrhea.**

A) I strongly agree B) I agree C) I do not agree

**3.4 Childhood diarrhea occurs only in Africa.**

**A)** I strongly agree B) I agree C) I do not agree

**3.5 A mixture of guava and 'masepo’ leaves is good for treating diarrhea at home**

A) I strongly agree B) I agree C) I do not agree

**3.6 Diarrhea in a child can rapidly lead to death if not properly managed.**

1. I strongly agree B) I agree C) I do not agree

**3.7 Diarrhea in children can be successfully managed at home without going to the hospital.**

1. I strongly agree B) I agree C) I do not agree

**3.8 Weaning can cause diarrhea in a child**

1. I strongly agree B) I agree C) I do not agree

**3.9 Teething can cause diarrhea in children***.*

1. I strongly agree B) I agree C) I do not agree

**SECTION C: PRACTICES ON CHILDHOOD DIARRHEA MANAGEMENT AND PREVENTION**

**4.1 How to you determine if a child has diarrhea? (Choose two answers).**

A) Measuring the mid-upper arm circumference

B) Measuring the child’s height

C) Asking the child’s age

D) Noting the level of dehydration in the child.

E) I don’t know

**4.2 Have you ever heard about oral rehydration salt?** A) YES B) NO

- 1. **Have you ever administered it to a child?** A) YES B) NO

**4.4 How will you prepare an oral rehydration salt (ORS) solution?**

1. Put 1 pack of ORS in 1liter of water
2. Put 1 pack of ORS in 1.5 liters of water
3. Put 1 pack of ORS in water
4. I have forgotten
5. I don’t know

**4.5 How much ORS/Sugar-salt solution per day will you give a 2-year-old child who has a diarrheal disease?**

A) 1liter B1/2 liter C) As much as the child can take D) 2 liters

**4.6 Have you ever heard about zinc supplements?** A) YES B) NO

**4.7a. Have you ever administered it to a child suffering from diarrhea?** A) YES B) NO

**4.8 Will you use Zinc Supplements to treat childhood diarrhea?** A) Yes B) No C) I don’t know

**4.9 If** Yes**, how long will you administer zinc supplements to a child suffering from diarrhea?**

A) 3 -5 days B) 1week C) 10- 14 days D I don’t know

- 1. **Have you ever heard about home-made Salt sugar solution? A**) YES B) NO

**4.11 a. Have you ever administered it to a child suffering from diarrhea?** A) YES B) NO

**4.11b What items will you advice a breastfeeding mother to use in preparing a salt sugar solution at home?**

1. 1teaspoon of salt, and 10 level teaspoons of sugar in 1 liter of clean water.
2. ½ teaspoon of salt, 5 cubes of sugar in 1 liter of clean water.

C) 1teaaspoon of salt, 5 cubes of sugar in 1.5liters of clean water.

D) ½ teaspoon of salt, 6 level teaspoon of sugar in 1 liter of clean water

E) I do not know

**4.12** **If a child has diarrhea, what treatments will you advise the mother to use? (Choose at most 3 answers)**

A) ORS only B) Zinc only C) Boiled herbs D) SSS (Sugar-Salt solution) only D) Water only E) Boiled paw-paw leaves E) Breast milk G) Nothing

F) Others………………

**4.13 If a child has diarrhea how often do you give him/her fluids?**

A) Less than usual B) Same as usual C) More than usual D) I don’t know

**4.14 What method of feeding will you advice a breastfeeding mother to use for a 3 months old baby suffering from diarrhea?**

**A)** Formula milk only B) No feeding, just mineral water (TANGUI, SUPERMONT)

C) Exclusive breastfeeding D) Juice or Yoghurt E) Others………………………………

- 1. **Which of the following should be done when a child has diarrhea?**

1. Continue breast feeding B) Stop breast milk C) Stop all feeding D) Give zinc tablets E) Give the child plenty to drink F) Avoid giving water G) Give antibiotics H) Give oral rehydration salt (ORS solution I) Do nothing J) I don’t know

**4.16 What other thing do you give a child suffering from diarrhea**

1. Charcoal, oil and salt
2. Palm oil
3. Squeezed guava leave juice
4. Palm kernel and guava leaves
5. None of the above
6. Others ………………………………………………….

**This completes the interview. Thank you very much for participating!!!**
