# Supplementary material for: Knowledge, attitude and practices of community health workers on managing and preventing childhood malaria and diarrhea in Fako Division, South West Region, Cameroon; A mixed method study
Source: PLOS Glob Public Health. 2023 Feb 21;3(2):e0001093. doi: 10.1371/journal.pgph.0001093 (PMC10021294; doi:10.1371/journal.pgph.0001093)
Supplement: S1 Text — (DOCX) [file pgph.0001093.s004.docx]

## Focus group discussion interview guide

**Knowledge, attitude and practices of community health workers on managing and preventing childhood malaria and diarrhea in Fako Division, South West Region, Cameroon; A mixed method study**

**SECTION A : SOCIO-DEMOGRAPHIC INFORMATION**

- What is your gender?
- How old are you?
- Which health area do you work for?
- How long have you worked as a community health worker?

**SECTION B: MAIN ROLE AS A CHW IN MANAGING CHILDHOOD DIARRHEA**

- What do you understand by childhood diarrhea?
- What causes diarhhea in children?
- How do you recognise a child who has diarrhea (signs and symptoms)?
- Will you advise the use of traditional medicine as treatment for a child suffering from diarrhea? Give reasons for your answer.
- What actions do you take when you recognise a child who has diarhhea?
- Do you know about ORS, If Yes, how is it prepared and administered to a child with diarhhea?
- Do you know about salt sugar solution, If Yes, how is it prepared and administered to a child with diarhhea?
- What other treatment do you give to a child who has diarrhea?
- What actions will you advise a breastfeeding mother to consider as preventive measures for childhood diarrhea?

**SECTION C: CHWs MAIN ROLE IN MANAGING CHILDHOOD DIARRHEA**

- What causes malaria?
- How is malaria transmitted
- What are the three main signs and symptoms of malaria in children?
- Do you think malaria is a disease of the poor?
- What do you do when you have a child with symptoms of malaria in your community?
- What are the common malaria treatments you give to children suffering from malaria in your community?
- Name 5 ways through which malaria can be prevented in your community.
- What is the first line treatment for malaria in Cameroon?
