# Supplementary material for: Knowledge, attitude and practices of community health workers on managing and preventing childhood malaria and diarrhea in Fako Division, South West Region, Cameroon; A mixed method study
Source: PLOS Glob Public Health. 2023 Feb 21;3(2):e0001093. doi: 10.1371/journal.pgph.0001093 (PMC10021294; doi:10.1371/journal.pgph.0001093)
Supplement: Attachment — Submitted filename: Response to reviewers.docx [file pgph.0001093.s005.docx]

Dear Editor,

The authors of this manuscript are pleased with the input of the reviewers. Please find attached the revised version of our manuscript PGPH-D-22-00391 for *PLOS Global Public Health Journal.* Responses to the comments have greatly improved the manuscript. We have also conducted additional analysis for the data.

The reaction to reviewers’ comments are as shown below (point-by-point reply):

**Editorial remarks**

1. Please provide an Author Summary. This should appear in your manuscript between the Abstract (if applicable) and the Introduction, and should be 150–200 words long. The aim should be to make your findings accessible to a wide audience that includes both scientists and nonscientists. Sample summaries can be found on our website under Submission Guidelines: https://journals.plos.org/globalpublichealth/s/submission-guidelines#loc-parts-of-a-submission Alternative link: <http://journals.plos.org/ploscompbiol/s/submission-guidelines#loc-authorsummary>

Response: The Author summary has been provided (between the ABSTRACT and the INTRODUCTION Section).

2. Reviewer #1:

ABSTRACT: There is no conclusion section for the abstract. Would divide Line 39 into Conclusion section

Response: The conclusion section has been divided as suggested (Abstract: Line 39)**.**

Line 58: That sentence is a little confusing. It would be good to clarify the definition of ‘the world’s 18th country’. Is that a ranking?

Response: Clarification has been made on the definition of ‘the world’s 18th country (Introduction section: Line 71)**.**

Reference #5 is also not appropriately written in the References Section so reader is unable to refer to the primary source of information to get a clearer understanding of the sentence.

Response: Reference #5 has been corrected in the Reference Section (INTRODUCTION Section: Line 72 and REFERENCE Section: Lines 667-669).

Line 61: Can remove ‘the diseases’ in the sentence as the listed diseases are self-explanatory.

Response: Suggestion applied (INTRODUCTION Section: Line 74).

Line 84: Assuming KAP refers to ‘Knowledge attitude and Practices’ but this is not alluded to in any section of the INTRODUCTION. This needs to be fully spelled out initially before abbreviating. Although there is a list of abbreviations at the end, it would be helpful to the reader to see the full word in the body of the paper initially and then made into an abbreviation when used again. Would check that this occurs for each abbreviation

Response: Suggestion applied (INTRODUCTION Section: Line 99).

Last paragraph references other studies that have done work on community healthcare workers and what they have reported. There indeed is also the lack of literature on childhood malaria and diarrhea in Cameroon. However, authors have not stated the AIMS of this study or the PURPOSE for conducting this study. There is also not a clear hypothesis for the research question being asked. The last paragraph should clarify the research question

Response: The Aim of this study has been stated in the last paragraph, a clarification has been made on the research hypothesis and the research question (INTRODUCTION Section; Last paragraph, Lines 95-97 and Lines 101-104).

METHODS:

Line 113: refer to HD before using the abbreviation. Study Design seems to be mixed-methods with quantitative portion described in analysis but qualitative component no clarified. This should be revisited by the authors to determine the appropriate methodology and study design as this seems to be more like a cross-sectional mixed methods study.

Response: Suggestion applied. The cross-sectional mixed methods study design has been written as suggested (Study design Section: Line 134) and the abbreviation has been written in full (Study Design Section: Line 140).

Line 116: good description for why this health district was left out

Response: A description has been made on the reason why this health district was left out (Study Design Section: Lines 144-145).

Line 118: Should provide description of Inclusion criteria? The subsection for “Criteria for Inclusion/Exclusion” does not detail the inclusion and exclusion criteria in detail. Would revisit this to help reader understand how participants were chosen What is the total number of participants in the study? What is the sample size or the sampling procedure for the study? Recruitment? How were certain participants excluded and why? The methods should go more into detail about the criteria used. This could be expressed as a figure as well.

Response: Inclusion and Exclusion criteria for this study has been provided under the Section Criteria for Inclusion/Exclusion (Line 183-186). The Sampling procedure and recruitment has been described in writing under the Section Study design (Lines 148 – 152)**.**

Line 156: there should be reference for the data analysis program for SPSS version 25 Quantitative analysis is described but the data from the focus groups is not explained on how themes were created from the discussion. Examples of types of analyses include reflexive thematic analysis? Deductive analysis? Was the thematic analysis done by one author or multiple? The methods should include the analysis of the Qualitative portion as these responses in the focus groups are a large part of the Results section

Response: Reference for the SPSS version 25 has been provided (Data Analysis Section: Line 193 and REFERENCE Section: Lines 720-722). A description has been made on how the data from the focus group was analyzed and an explanation has been given on the authors who did the analysis of the data from the focus group discussion (Data Analysis Section: Lines; 210 – 214).

Lines 162-163: would be beneficial to understand the scores if described further in detail. Would revisit the analysis and input p-values when discussing the associations between outcome and variables. The analyses need more description.

Response: A description has been made on how the scores were used to determine the level of knowledge, attitude and practices (Data Analysis Section: Lines 193 – 196 and Line 200). Data analysis has will be revisited and the p-values provided (Lines 207-208).

RESULTS

Sociodemographic characteristics would be well displayed as a table rather than in a paragraph. This would help the reader easily refer to this and also improve word count.

Response: Sociodemographic data has been displayed as a table (Page 11 and Page 12) and a reduction has been made on the word’s description as suggested (Lines 217-2018, Lines 222-225 and Line 227-229)

First sentence seems incomplete: “Only of the 143 CHWs work in Fako…” Unsure if there is supposed to be a number after the first word.

Response: The sentence has been corrected (Lines 217).

Line 185-187: the first sentence is confusing. “composed of 9 participants each 11 participants were involved.” Would clarify more with this statement.

Response: The sentence has been corrected (Line 227-228).

Would recommend either shortening the statements added from the focus groups or make a supplemental figure for the responses so reader can refer to them. The addition of the full responses prolongs the wording of the paper and can be confusing at times.

Response: Statements from the responses of the focus group discussions have been shortened as suggested (RESULTS Section; Lines 340, 345, 347, 418,423, 426-427 and 446-449).

DISCUSSION:

Paragraph starting on line 539, first sentence is a repeat of the prior paragraph. This should be cross-sectional mixed methods

Response: The sentence has been corrected (Line 595-598), and the cross-sectional mixed methods has been used as suggested.

OVERALL: Manuscript will need to provide further information regarding its methodology and analyses. This was noted to be a cross-sectional study in the Methods section, however, the addition of qualitative data and themes according to the results section will need to be described further. The presentation of the data in the Results section should be further expanded as the body of the Results section is very wordy. The use of demographic figures or tables and other forms of representation of the qualitative data should be explored.

Response: Information on methodology and data analysis has been provided with correction made on the results of the Spearman Correlation analysis (ABSTRACT Section: Lines 37-40; RESULTS Section: Lines 374-375; Table 6 Page 23; Lines 485 – 486 and Table 10 Page 32). The cross-sectional mixed method study design has been written as suggested. Tables have been added to present data in the Results section (Table 1 Page 11 and Table 2 Page 12).

Reviewer #2: Summary and Overall recommendation

The main research question is whether there is a correlation between the level of knowledge, attitudes and practices of community healthcare workers in the prevention and management of childhood malaria and diarrhea in the Fako Division, South West Region, Cameroon. The study results and data analysis show a positive correlation between a higher level of knowledge which influences a more positive attitude and use of the correct practices in the prevention and management of childhood malaria and diarrhea in line with the country’s and WHO guidelines (lines 534-54, page 37).

The conclusion of the study is that amongst the study population, more than 50% of the 80CHWs who participated in the study had good knowledge and more than 60% had negative attitudes and poor practices on childhood malaria management and prevention.

The conclusion on childhood diarrhea management and prevention showed similar results where more than 50% of the CHWs had poor knowledge, 75% had negative attitudes and more than 75% used good practices. There were interesting conclusions on the preference for traditional herbal concoctions in the treatment of both childhood malaria (75%) and diarrhea (33%) which is in line with the correlation findings on the knowledge, practices and attitudes discussed above. The research fits in with existing literature in the region with references drawn from Nigeria, Rwanda and Kenya.

Strengths of the manuscript are on the study design, data tools, collection and analysis of the data and description of the results. The selection of the Chi Square and Spearman Correlation tests was appropriate. The study setting discussion section could be strengthened with more details as highlighted in the section on major issues below. Overall this is a good study on Knowledge, Attitude and Practices of CHWs on management and prevention of childhood malaria and diarrhea as a first of its kind in Cameroon and is recommended for publication following requested revisions.

Major Issues

I. Study Setting: Include a description of the community health care system in Cameroon, in particular how the CHWs are linked to the health centers, health areas and district.

Response: A description has been made on the community health care system in Cameroon (Study Setting Section: Lines 124-132).

Also include the number of health centers that the CHWs serve or refer patients to in the 3 health districts selected and whether these are only public or private facilities. This is important in creating a better context towards understanding the CHWs operating environment.

Response: The number of health centers for referrals in each of the 3 health districts has been provided and explanation has been made on whether these health centers are public or private (Study Design Section: Lines 148 – 152).

Discussion: • Line 474, kindly give examples of the other disease control programs that CHWs are trained on.

Response: Examples of other disease control programs has been provided (DISCUSSION Section: Lines 520-521

• Line 476, kindly include more detail on what the minimum package of activities are for CHWs in the region.

Response: Information on minimum package of activities has been provided (DISCUSSION Section: Lines 524-527).

• Line 487, discussion only focuses lack of resources on negative attitudes by CHWs towards malaria management and prevention; what about the influence of low knowledge or lack of access to trainings as mentioned earlier.

Response: Suggestion applied (DISCUSSION Section Lines 537-538).

• Line 499 to 501, the comparison with Nigeria on the practice of using herbs in treatment of malaria shows quite a significant difference with Cameroon at 75% and Nigeria at 37% recommend that authors look for a country/ study with statistics with a closer similarity in results for better comparison.

Response: Studies from Senegal and Nigeria have been used to make comparisons as suggested (DISCUSSION Section: Lines 552-558; Lines 586-587 and the REFERENCE Section: Lines 733-744)

III. Literature references can be strengthened by drawing more examples from studies in the West Africa region as opposed to East Africa as the endemicities and cultural practices vary slightly. E.g. line 529 pulls a reference from Indonesia in South East Asia.

Response: Other studies from Senegal and Nigeria have been used as suggested (DISCUSSION Section: Lines 552-558; Lines 586-587 and REFERENCE Section: Lines 733-744).

Response: A statement has been made on the availability of data for this study (Availability of data and materials Section: Line 641). The data has been provided as Supporting Information files for this paper (LEGEND; Supporting Information, where S1 Table. Data set (XLS) is data from the questionnaires and S1 Appendix. Table of themes (DOCX) is data from the FGDs: Line 751-753).

After these changes, we hope that the manuscript is now suitable for publication in your journal.

With best regards,

Ndum Naomi Chi, Raymond Babila Nyasa and Akoachere Jane-Francis
