# Supplementary material for: Knowledge, attitude and practices of community health workers on managing and preventing childhood malaria and diarrhea in Fako Division, South West Region, Cameroon; A mixed method study
Source: PLOS Glob Public Health. 2023 Feb 21;3(2):e0001093. doi: 10.1371/journal.pgph.0001093 (PMC10021294; doi:10.1371/journal.pgph.0001093)
Supplement: Attachment — Submitted filename: Response to reviewers.docx [file pgph.0001093.s006.docx]

Dear Editor,

Thank you for giving us the opportunity to submit a revised draft of our manuscript. We appreciate the time and effort that you and the reviewers have dedicated to providing your valuable feedback on our manuscript. We are pleased with the input of the reviewers. Please find attached the revised version of our manuscript PGPH-D-22-00391 for *PLOS Global Public Health Journal.* We have been able to incorporate changes to reflect most of the suggestions provided by the reviewers. Responses to the comments have greatly improved the manuscript.

The reaction to reviewers’ comments are as shown below (point-by-point reply):

**Editorial remarks**

1. Please amend your detailed Financial Disclosure statement. This is published with the article. It must therefore be completed in full sentences and contain the exact wording you wish to be published.

a. State the initials, alongside each funding source, of each author to receive each grant.
b. State what role the funders took in the study. If the funders had no role in your study, please state: “The funders had no role in study design, data collection and analysis, decision to publish, or preparation of the manuscript.”

**Response:** Thank you for pointing this out. We have stated the Financial Disclosure statement in a full sentence that contains the exact wordings the authors wish to be publish (ACKNOWLEDGEMENT, Funding Section: Line 595).

The study was self-funded.

**Reviewer #3:**

Comment 1. My main concern is that this (2 in 1) manuscript is too lengthy. This is acceptable as it covers two different diseases (malaria and diarrhoea); however, the content can be shortened substantially, particularly results section. I suggest the following:

1.1.Combining qualitative results on KAP towards childhood malaria in one section rather than reporting them in the relevant separated 3 sections for each component of KAP. Similarly, the FGDs on childhood diarrhoea can be combined in one section. The content of these suggested sections should be substantially shortened to focus on important findings and responses only that will be useful for policymakers. The results are provided in the additional file.

**Response:** Thank you for pointing this out. However, the manuscript focuses on two different diseases that is why unfortunately the manuscript is too wordy and lengthy. For each of the diseases, quantitative and qualitative information for knowledge was grouped together and the same thing was done for attitude and practices. We believe that grouping KAP for qualitative data together for each disease would not have reduced the number of words in the manuscript. We have accordingly revised the manuscript and made it shorter by six pages. In addition, the important findings useful for policymakers have been stated out in the conclusion (CONCLUSION Section: Lines 557 - 564).

**Comment 2:** Text of results on knowledge, attitude and practices should be reduced substantially as there are high number of tables and figures displaying the results.

**Response:** Thank you for your suggestion. We agree with this and have incorporated your suggestion. The wordings on results have been reduced.

**Comment 3:**.Lines 215-219: these sentences can be removed. Also remove the subsection title (line 213). Add a statement that the general characteristics of the participants are shown in Table 1. Add the mean (SD) age of the 80 participants to first sentence (line 214).

**Response:** Thank you for this suggestion. The sentences have been removed including the subsection title. A statement has been added (Page 10, lines 218 - 219) to describe the general characteristics of the participants including information about the mean age (SD).

**Comment 4:** The questionnaire can be provided as a supplementary file. Many researchers will be interested in adopting this comprehensive pre-tested questionnaire.

**Response:** Agree. We have submitted the questionnaire and FGD interview as supplementary files alongside this reviewed manuscript.

**Comment 5:** The manuscript needs English editing by a native speaker to correct several spelling, punctuation and grammar mistakes in the text, tables and figures.

**Response:** You have raised an important point here. We have corrected spelling and grammatical errors throughout the manuscript.

SPECIFIC COMMENTS (Arranged according to text flow)

**Comment 6:** Line 19: in Fako Division, South West Region, Cameroon.

**Response:** We agree with this and have incorporated your suggestion on the title of the manuscript (TITLE Section: Lines 2 – 3, ABSTRACT Section: Line 19).

**Comment 7:** Line 20: “to 80 CHWs”

**Response:** Thank you for pointing this out. The sentence has been corrected.

**Comment 8:** Line 22: please rephrase to improve clarity.

**Response:** Agree. **Accordingly, w**e have modified the sentence (ABSTRACT, Methods Section: Lines 21 - 24).

**Comment 9:** Apply first appearance rule to the entire manuscript. For example use the abbreviation (KAP) of knowledge, attitude and practices throughout the text. Using CHWs, FGDs and other abbreviations were missed several times in different sections.

**Response:** Thank you for pointing this out. We have applied the first appearance rule on the abbreviations; KAP, CHWs, FGD, LLITN used on the entire manuscript.

**Comment 9:** Line 24: “The Spearman rank Correlation …….. between them.” This sentence can be removed (merge with previous sentence).

**Response:** We agree with this and have incorporated your suggestion by merging this sentence with the previous one (ABSTRACT, Methods Section: Lines 21 - 24).

**Comment 10:** Lines 38-39: “……. and attitude with practices (r = 0.257) on childhood malaria.” This long sentence can be split into 2 sentences to improve clarity.

**Response:** We agree with this comment. we have made some modifications by removing some of the results reported on the abstract so that the number of words can suit the submission guidelines of the journal.

**Comment 11:** Line 40: “KAP …… were generally poor.” But 52.5% of them had good knowledge about malaria and 82.25% carried out good practices on management and prevention of diarrhea.

**Response:** Thank you for pointing this out. We agree with this comment. We have modified the conclusion stating CHWs have inadequate knowledge on childhood malaria and carry out good practices on childhood diarrhea management (ABSTRACT, Conclusion Section: Lines 33 - 37).

**Comment 12:** Line 79: “community health workers (CHWs)” and use the abbreviation CHWs in line 81 and many other sentences, e.g. line 137,145, 146, etc.

**Response:** Thank you for pointing this out. We have applied the first appearance rule on the abbreviations CHW throughout the manuscript.

**Comment 13:** Line 90: “…. have inadequate knowledge …”

**Response:** Thank you for this suggestion. We have made the correction as suggested (INTRODUCTION Section: Line 91).

**Comment 14:** Line 95: “….between socio-demographic factors (such as ???) and CHWs’ …”

**Response:** Thank you for pointing this out. I/We agree with this
comment. We have made modification on the sentence and stated a socio-demographic factor associated with CHWs’ knowledge (INTRODUCTION Section: Line 96)

**Comment 15:** Line 97: “…in Africa, few studies …”

**Response:** Thank you for raising this point. The manuscript has been revised to include Kenya, Zambia , Rwanda, Uganda, Somaliland, Cameroon, Nigeria and Senegal where and when this studies were conducted (INTRODUCTION Section: Lines 98 - 99) .

**Comment 16:** Lines 95-102: try to avoid much repetition of “CHWs’ knowledge, attitudes and practices”, “management and prevention of childhood malaria and diarrhea” in these sentences.

**Response:** We agree with this and have incorporated your suggestion throughout the manuscript.

**Comment 17:** Line 143: “A pretested structured questionnaire …” But it is stated as a semi-structured one in abstract (line 20). Please revise and indicate as pretested in abstract.

**Response:** Thank you for pointing this out. We have effected the correction using ‘pre-tested’ questionnaire (ABSTRACT Section: Line 20).

**Comment 18:** Line 177: spell out ORS here.

**Response:** Thank you pointing this out. We have spelt ORS in full (MATERIALS AND METHODS, Questionnaire Administration Section: Line 181).

**Comment 19:** Line 178: change to “long lasting insecticide treated mosquito nets (LLINs)”.

**Response:** Thank you for pointing this out. We have applied the suggestion (MATERIALS AND METHODS, Questionnaire Administration Section: Line 182).

**Comment 20:** Line 194: Knowledge score, attitude score and practice scores. Rephrase.

**Response:** We agree with this comment. Therefore, we have corrected the sentence accordingly (MATERIALS AND METHODS, Data analysis Section: Line 198).

**Comment 21:** Table 1: add (n = 80) at the end of table’s title.

**Response:** Thank you for this suggestion. We have added a number (n = 80) at the end of the title as suggested (RESULTS Section: Table 1, Page 10).

**Comment 22:** Table 1: revise age group first class; aged 21 years and above is one of inclusion criteria (line 180). Add (years) after Age group, and remove “years” from all groups.

**Response:** Thank you for pointing this out. We have revised the variable ‘Age group’. The first class for age group has been corrected and “years” has been removed from all age groups. (RESULTS Section: Table 1, Page 10 - 11).

**Comment 23:** Table 1: Remove the rows of “total” for gender, “Mean age (±SD)” for age group, and “Mean (±SD)” for No. of children < 5 years.

**Response:** Thank you for this suggestion. We have incorporated your suggestion by removing the rows with “total” from the following variables; gender, age group and No. of children (RESULTS Section: Table 1, Page 11).

**Comment 24:** Table 1: Add rows for health districts (3 groups).

**Response:** Thank you for pointing this out. We agree with this
comment. On Table 1, we have added three rows under the variable ‘health districts’ (RESULTS Section: Table 1, Page 11)**.**

**Comment 25:** Table 1: Regroup no. of children 2).

**Response:** Thank you for your suggestion. We have revised the grouping of the categories for the variable ‘number of children’ on Table 1 (Page 11).

**Comment 26:** Table 1: the total of “Other Occupation” is 100 and not 80. Please revise.

**Response:** Thank you for pointing this out. We have applied the correction on total for the variable “Other Occupation” (RESULTS Section: Table 1, page 11).

**Comment 27:** Correct Table 1 numbering for FGDs to Table 2.

**Response:** Thank you for pointing this out. We have made the correction for the numbering of Table 2 (Page 12).

**Comment 28:** Table 2: add (n = 29) at the end of table’s title.

**Response:** Thank you for this suggestion. We have added a number (n = 80) at the end of the title as suggested (RESULTS Section: Table 2, Page 12).

**Comment 29:** Table 2: Remove the row of “total” for gender

**Response:** Thank you for this suggestion. We have incorporated your suggestion by removing the rows with “total” from the following gender (RESULTS Section: Table 2, Page 12).

**Comment 30:** Table 2: Add (years) after Years of experience, remove “years” from all groups, and merge the last 2 groups as > 15.

**Response:** Thank you for your suggestion. We have applied your suggestion by adding ‘years’ after the variable ‘Years of experience’ and we have merged the last two categories of the variable (RESULTS Section: Table 2, Page 12).

**Comment 31:** Table 4: Insects that because malaria get the infection from. This is unclear.

**Response:** Thank you for pointing this out. We have applied the correction (RESULTS Section: Table 4, page 16).

**Comment 32:** Line 311: LLINs

**Response:** We agree with this comment and have incorporated your suggestion on lines 306 of the manuscript.

**Comment 33:** Line 459: add more one which groups (education and district) had poor/negative or good/positive level.

**Response:** Thank you for pointing this out. It would have been
interesting to explore this aspect. However, in Cameroon the right to education is well respected. The educational system shows no discrimination for populations in different areas. This is also what was also observed throughout the study.

**Comment 34:** Line 590: LLINs: long lasting insecticide treated mosquito nets

**Response:** We agree with this comment and have incorporated your suggestion on line 579 of the manuscript.

**Comment 35:** The format of the list of references is not standard and not according to journal’s style. It should be prepared using proper reference management software or following a PDF file of any article recently published in the journal.

**Response**: Thank you for pointing this out. We agree with this comment. We have revised both the citation and references format (REFERENCES Section: Line 605) used on the entire manuscript.

Reviewer # 4

TITLE:

**Comment 1:** “Knowledge attitude and practices of community health workers on the management and prevention of childhood malaria and diarrhea in Fako Division, South West Region, Cameroon”

Consider adding a comma before “attitude”

**Response:** Thank you for pointing this out. We have made the correction (TITLE Section: Line 1).

**Comment 2:** Consider “Knowledge, attitudes and practices of community health workers on managing and preventing childhood malaria and diarrhea in Fako Division, South West Region, Cameroon: A mixed methods study”

**Response:** Thank you for your suggestion. We agree with this
comment. We have, accordingly revised the title of the manuscript (TITLE Section: Line 1 - 3).

ABSTRACT:

**Comment 3:** Under methods, consider adding “s” to CHW, and indicating the number of focus group discussion (line 21”

**Response:** Thank you for pointing this out. As you suggested, we have made this correction on line 21 of the manuscript.

INTRODUCTION

**Comment 4:** The introduction appears to lack similar studies conducted outside of Cameroon. The authors should consider strengthening the introduction with related studies in Africa, if available.

For example, the authors indicated in lines 96-99:

“Although much research has been done on childhood diarrhea and malaria in Africa, very few studies have evaluated CHWs knowledge attitude and practices (KAP) on management and prevention of childhood malaria and diarrhea in Cameroon particularly as many cases still report in hospitals in the study area.”

Citing relevant studies to back the above statement may be helpful. Some of the studies cited in the discussion may be helpful here.

**Response:** Thank you for raising this point. The manuscript has been revised to include Kenya, Zambia , Rwanda, Uganda, Somaliland, Cameroon, Nigeria and Senegal where and when this studies were conducted (INTRODUCTION Section: Lines 98 - 99).

METHODS

**Comment 5:** Lines 143-145: “A pretested structured questionnaire was administered to both male and female CHWs who met the inclusion criteria. From this population simple random sampling was used to select community health workers for focus group discussion.”

**Response:** Thank you for pointing this out. We have made modification on the methods (Lines 151 - 153) and have deleted the sentence.

**Comment 6:** Lines 182-183 has the information: “The FGDs were composed of randomly selected CHWs who already completed the questionnaire.”

It seems unclear why random sampling was used for selecting focus group discussion participants. Did the authors mean survey? We typically do not use randomization to select qualitative study participants.

**Response:** Thank you for pointing this out. We agree with the comment. We revised the manuscript and made correction by stating that convenient sampling was used to recruit participants for the FGDs (MATERIALS AND METHODS Section: Line 149 – 150, line 186).

**Comment 7:** Lines 206-210: Authors should consider referencing “reflective thematic analysis”, and providing the steps they took to conduct the thematic analysis. Information available in lines 206-210 seems scanty.

**Response:** Agree. Accordingly, we have modified this on these lines 211 – 215, under the section Data Analysis.

**Comment 8:** Under data analysis, the authors should consider assessing at least the reliability (by calculating Cronbach alpha) of the following constructs:

a) Knowledge on management and prevention of childhood malaria
b) Attitudes on management and prevention of childhood malaria
c) Practices regarding management and prevention of childhood malaria
d) Knowledge on management and prevention of childhood diarrhea
e) Attitude score on management and prevention of childhood diarrhea
f) Practices regarding management and prevention of childhood diarrhea

The results of such analyses could determine whether the questionnaire items may be relevant for subsequent studies or could be improved (by eliminating some items) to help reduce the burden on respondents.

**Response:** Thank you for this suggestion. It would have been interesting to explore this aspect. In order to use Cronbach’s alpha as a reliability coefficient, the data from the measures must satisfy condition of [Independence between errors](https://en.wikipedia.org/wiki/Independence_(statistics)). In this study, this condition is not fulfilled to conduct the Cronbach’s alpha analysis.

From the authors’ perspective concerning the management of diarrhea in Cameroon, we believe that the management and prevention of these diseases are dependent on each other. For management of malaria for example, it is required that even before administering malaria treatment; the patient should be provided an LLIN to prevent further malaria infection. However, if it were possible, from the reviewers comment the analysis would have help to ascertain or improve the quality of the questionnaire.

RESULTS

Table 2

**Comment 9:** Under years of experience, consider making the following edits:

Space between “5, 10, and 20” and years.

**Response:** Thank you for your suggestion. We agree with this
comment. We have effected the correction considering comments from other reviewers. We added ‘years’ after the variable ‘Years of experience’ and merged the last two categories of the variable (RESULTS Section: Table 2, Page 12).

**Comment 10:** There appears to be a problem with 0-5 years, and 5-10 years. If a participant had 5 years of experience, which category of years of experience was the participant likely to be placed?

**Response:** Thank you for pointing this out. We agree with this
comment. We have made revisions on the lower limits of the different categories. The authors considered ages reported as a full year for the upper limit of each category (RESULTS Section: Table 2, page 12).

Table 5

**Comment 11:** RDT should either be spelled in full in the table or it should be defined at the end of the table.

**Response:** Thank you for your suggestion. We have effected the correction by spelling RDT in full (RESULTS Section: Table 5, Page 19).

DISCUSSION

**Comment 12:** Authors should consider including the limitations of their study as a section just before the conclusion.

**Response:** Thank you for raising this very important concern. We have included a section on manuscript with the limitations of the study (LIMITATION Section: Line 566 - 570).

**Comment 13:** There are some issues with formatting, with some statements in italics when this should not be the case (see lines 516, 520-522, 550-551). Authors should consider addressing this issue.

Also, throughout the text, authors should consider checking their capitalizations.

**Response:** Thank you for pointing this out. We agree with this and have incorporated your suggestion on lines 501, 505, 506, 535 and 536 of the manuscript.

In addition to the above comments, spelling and grammatical errors pointed out by the
reviewers have been corrected throughout the manuscript.

Reviewer # 5

**Comment 1:** Recommendation to review the grammar and spelling, there are some errors that can be addressed easily.

**Response:** Thank you for raising this concern**.** We agree with this and have incorporated your suggestion**. S**pelling and grammatical errors pointed out by the
reviewers have been corrected throughout the manuscript.

**Comment 2:** Recommendation to state Fako Division, South West Region of Cameroon instead of Fako Division in the abstract.

**Response:** Thank you for your suggestion. Considering the comments from the other reviewers, we have incorporated your suggestion by revising the title fo the manuscript (ABSTRACT Section: Line 19).

**Comment 3:** State the number of CHWs involved in Focus Group Discussions. Correct CHW to CHWs

**Response:** Thank you for pointing this out. We have agree with this comment. The numbers CHWs involved in FGDs was already stated under the study design. However, the suggestion has been applied under the “Criteria for Inclusion/Exclusion” on the manuscript (Line 186).

**Comment 4:** In the results and discussion provide the correct answer for the reader as opposed to stating the percentage of the respondents who selected each of the options for the question.

**Response: Agree.** We have revised the Results section of the manuscript as suggested. Only the correct answers are now reported under the results and the discussion.

**Comment 5:** According to Boston University School of Public Health, [The Correlation Coefficient (r) (bu.edu)](https://sphweb.bumc.bu.edu/otlt/MPH-Modules/PH717-QuantCore/PH717-Module9-Correlation-Regression/PH717-Module9-Correlation-Regression4.html#:~:text=The%20sample%20correlation%20coefficient%20(r,for%20accumulated%20saving%20over%20time.) 0.2-0.4 is classified as weak positive association, the authors state association between knowledge and attitude and practices on childhood diarrhea is classified as a positive significant association (with r values of 0.265 and r of 0.329). Please provide the p values.

**Response:** Thank you for raising this concern. We agree with your comment. On line 344 – 345; Table 6 (Page 21) and on lines 438 – 439; Table 10 (page 30 ). We have reported the P values from the analysis.

**Comment 6:** Consider revision of the conclusion statement of abstract. In the results, 52.5% of the participants were stated to have good knowledge on management and prevention of childhood malaria, however it was stated that knowledge, attitudes and practices of CHWs on childhood malaria….were generally poor. Perhaps a revision that the level of knowledge was inadequate.

**Response:** Thank you for pointing this out. We have, accordingly, revised the conclusion to lay emphasize this point both on the abstract and the main write-up (ABSTARCT Section: Lines 33 – 37, CONCLUSION Section: Lines 557 - 564).

**Comment 7:** In author’s summary, suggestion to include KAP survey and focus group discussions for the reader to understand the methods used to gather data.

**Response:** Thank you for your suggestion. We have applied your suggestion. The KAP survey and FGDs have been included added to the author’s summary (AUTHOR SUMMARY Section: Line 50 - 52).

**Comment 8:** In the transcript where revisions were highlighted, on pgs 26-28 there were amendments to the quotes (parts removed) made by FGD participants. Please clarify the reasons for this and review if the amendments affected the content of the quote.

**Response:** Thank you for raising this concern. We agree with your comment.

However, we believe that the words removed from participant’s responses in the FGDs did not affect the content. In addition, on the first manuscript the authors reported similar responses from more than one participants from each district. Based on the reviewers’ comment on the length of the manuscript similar responses from the same health district where removed where necessary, leaving just one respond from each district.

**Comment 9:** Introduction – provide context for the reader with the following information

- Ratio of Health care workers to population; ratio of community health workers to population
- Ratio of health facilities to population.
- Qualifications or training required to become a community health worker.

**Response:** Thank you for your suggestion. We agree with this comment. The following information with references has been provided as suggested;

Health care workers to population ratio (Line 73 )

Community health worker to population ratio in Fako division (Line 74 -75).

Health facility to population ratio in Fako division (Line 75-76).

Information on the qualification, selection and training of CHWs (Lines 84 - 86).

**Comment 10:** Page 4 – provide additional information on the recent studies regarding incomplete knowledge.

**Response** Thank you for raising this point. The manuscript has been revised to include Kenya, Zambia , Rwanda, Uganda, Somaliland, Cameroon, Nigeria and Senegal where and when this studies were conducted (INTRODUCTION Section: Lines 98 - 99).

**Comment 11:** It does not state where these studies were conducted or when or any information on the results.

**Response:** Thank you for raising this point. The manuscript has been revised to include Kenya, Zambia , Rwanda, Uganda, Somaliland, Cameroon, Nigeria and Senegal where and when this studies were conducted (INTRODUCTION Section: Line 98 - 99).

**Comment 12:** Page 6 – line 122 – Are there any statistics on the increased access to and utilization of services by the target population? What is defined as the target population?

**Response:** Thank you for your suggestion. We have provided more information on the utilization of services by populations in hard to reach areas (INTRODUCTION Section: Lines 124 - 127). A revision has been made on the definition of target population (INTRODUCTION Section: Line 124).

**Comment 13:** Page 6 – line 135 – Clarify what is meant by the chiefs of center.

**Response:** Agree. We have revised the sentence and clarification has been made on the meaning of chief of center (INTRODUCTION Section: Line 139)**.**

**Comment 14:** Page 7 – line 144 – were participants invited, selected or randomly selected to participate in the FGDs. On page 9 line 182 of the transcript the authors state the FGD participants were randomly selected from those who completed the survey.

**Response:** Thank you for pointing this out. We agree with the comment. We revised the manuscript and made correction by stating that convenient sampling was used to recruit participants for the FGDs (MATERIALS AND METHODS, Focus Group Discussion: Line 150).

**Comment 15:** Page 8 - Expand the description of the design of the survey; what studies/ surveys were referred to; state how many questions were in each section; if participants were able to select more than one option for some questions or not; state the language(s) the survey was administered; who administered the survey; if the survey participants received an honorarium or not. How were the participants contacted for the FGDs? Was the training delivered after the FGDs were held or after the survey was administered? Were the FGDs organized with senior personnel and junior personnel together or separately? In what language(s) were the FGDs conducted? Who led the FGDs?

**Response:** Thank you for your suggestion. We agree with this comment. We have provided more information the sections; Study design (Lines 151 - 153) and Questionnaire Administration (Lines 165), explaining the methods employed in this study. In this study, the FGDs were conducted in English language facilitated by the researcher. During the FGDs, the researcher trained the CHWs on their roles in the management of these diseases.

**Comment 16:** Page 8 – Line 177 – Spell out ORS for the first time use

**Response:** Thank you for pointing this out. We have applied the correction by spelling out ORS (MATERIALS AND METHODS, Questionnaire Administration Section: Line 181).

**Comment 17:** Page 8 – Although open ended questions were mentioned for the questionnaire, it is unclear how these responses were analyzed or which questions were open ended questions.

**Response:** Thank you for raising this point as it is a very important comment. From your concern, we have made modification on line 165, stating that we pre-tested a questionnaire with multiple-choice questions. This is because the authors chose to ignore the responses from the open-ended questions. From your concern we also noticed and corrected the number of questions on knowledge (line173), attitude (line 176) and practices on line 180 .

**Comment 18:** Page 10 – line 214 – 143 CHWs, 80 participated. On the transcript this is reported as 84.2%. 80/143 as a percentage is 55.9%.

**Response:** Thank you for pointing this out. We agree with this comment. Considering comments from other reviewers, we have modified this section by making a statement concerning the general characteristics of the participants on line 219.

**Comment 19:** Page 10 – Table 1 – is it possible to show the breakdown of length of service by education, and level of education by gender.

**Response:** Thank you for pointing this out. It would have been
interesting to explore this aspect. However, in Cameroon the right to education is well respected. The educational system shows no discrimination males and females. This is also what was also observed throughout the study as the level of education was uniformly distributed in the different health district.

**Comment 20:** Page 11 – Table 1 – what is meant by unemployed? Are CHWs employed by the government and do they receive a salary? Or were unemployed and employed CHWs included in the study?

**Response:** Thank you for raising this point. In this study, unemployment referred to having another job aside of being a community health worker. This was explained to the CHWs during questionnaire administration.

**Comment 21:** For all tables it would useful to highlight to the correct answer(s) and to state what is the correct answer in the narrative.

**Response:** Thank you for your suggestion. We have applied the applied this suggestion by revising the results of the manuscript wherein only the correct answers are now reported in text under the results.

**Comment 22:** Page 13 – Line 239-240 – It is unclear if ACT is the first line treatment only or if others are also first line treatments. Spell out ACT the first time it is used.

**Response:** Thank you for your suggestion. We have applied the applied this suggestion by revising the results of the manuscript wherein only the correct answers are now reported in text under the results. We have also applied the first appearance rule applied on the entire manuscript (Table 3, Page 14).

**Comment 23:** Page 13 – Table 3 – spelling error – clothe

**Response:** Thank you for pointing this out. We have made this correction (Table 3, Page 13).

**Comment 24:** Page 14 – Table 3 – is something missing in the sentence – Every suspected malaria case should do and RDT?

**Response:** Agree. We have made the correction by adding ‘an’ to the sentence (Table 3, Page 14).

**Comment 25:** Page 14 – Table 3 – arrange duration of treatment in ascending or descending order.

**Response:** Thank you for this suggestion. We agree with this comment. We have made revision on the categories under the duration of treatment by arranging it in ascending order (Table 3, page 14).

**Comment 26:** To include in limitation that the questionnaire did not include a specific question on previous training on childhood malaria and diarrhea.

**Response:** Thank you for raising this very important concern. We have included this as one of the limitations of the study (LIMITATION Section: Line 566 - 570).

**Comment 27:** Page 17 – Table 4 – unclear statement “Insects that because malaria get the infection from”

**Response:** Agree. We have corrected error on the statement (Table 4, Page 16).

**Comment 28:** Page 19 – Table 5 – some formatting issues and is the statement “when I am chanced” accurate?

**Response:** Thank you for pointing this out. We agree with this comment. We have corrected the format of Table 5 and have also modified that statement noted (Table 5, Page 19).

**Comment 29:** Page 20 – Table 5 – under dose of antimalarial drugs administered in a day to a child, there is 1, 2, 3, and 5. Was there a 4?

**Response:** Thank you for raising this concern. No, there was no option for 4 doses.

**Comment 30:** The discussion can be strengthened by including any available data and reports on the type and frequency of training delivered to CHWs on childhood malaria and diarrhea; the availability and cost of malarial and diarrhea medication in Cameroon and particular in the Fako Division.

**Response:** Thank you for your suggestion. We have added more information about the training of CHWs (DISCUSSION Section: Line 468 - 470) and have also provided the definition for NGO under the line of abbreviations (580).

In Cameroon, Training is usually done by staff from the Ministry of Public Health, and local and international non-governmental organizations (NGOs), often before health campaigns and MDA programs and not regularly.

The aim of the study was to access CHWs’ KAP and hence very little emphasis was laid on the cost of malaria and diarrhea medication since CHWs are not expected to get these medications out of pocket payment.

**Comment 31:** Page 36 – reference is made to the significant association between level of education and health district with practices on childhood diarrhea management. It would be useful for the reader to have a graph to show the difference in practices across level of education and health district.

**Response:** Thank you for pointing this out. It would have been
interesting to explore this aspect. However, in Cameroon the right to education is well respected. The educational system shows no discrimination for populations in different areas. This is also what was also observed throughout the study.

In addition to the above comments, we have made some modifications by removing some of the results reported on the abstract so that the number of words can suit the submission guidelines of the journal.

After these changes, we hope that the manuscript is now suitable for publication in your journal.

With best regards,

Ndum Naomi Chi, Raymond Babila Nyasa and Akoachere Jane-Francis
